# QTL analysis of modifiers for pigmentary disorder in rats carrying *Ednrb*^*sl*^ mutations

**DOI:** 10.1038/srep19697

**Published:** 2016-01-22

**Authors:** Jieping Huang, Ruihua Dang, Daisuke Torigoe, Anqi Li, Chuzhao Lei, Nobuya Sasaki, Jinxi Wang, Takashi Agui

**Affiliations:** 1 College of Life Sciences, Northwest A&F University, Yangling, Shaanxi, China; 2 College of Animal Science and Technology, Northwest A&F University, Yangling, Shaanxi, China; 3 Laboratory of Laboratory Animal Science and Medicine, Department of Disease Control, Graduate School of Veterinary Medicine, Hokkaido University, Hokkaido, Japan; 4 Laboratory of Laboratory Animal Science and Medicine, School of Veterinary Medicine, Kitasato University, Aomori, Japan; 5 Division of Microbiology and Genetics, Center for Animal Resources and Development, Kumamoto, Japan

## Abstract

Pigmentary variation in animals has been studied because of its application in genetics, evolution, and developmental biology. The large number of known color loci provides rich resource to elucidate the functional pigmentary system. Nonetheless, more color loci remain to be identified. In our previous study, we revealed that two different strains, namely, AGH rats and LEH rats, but which had the same null mutation of the *Ednrb* gene (*Ednrb*^*sl*^) showed markedly different pigmented coat ratio. This result strongly suggested that the severity of pigment abnormality was modified by genetic factor(s) in each strain. To elucidate the modifier locus of pigment disorder, we carried out whole-genome scanning for quantitative trait loci (QTLs) on 149 F_2_ (AGH-*Ednrb*^*sl*^ × LEH-*Ednrb*^*sl*^) rats. A highly significant QTL, constituting 26% of the total pigmentation phenotype variance, was identified in a region around *D7Got23* on chromosome (Chr) 7. In addition, investigation on epistatic interaction revealed significant interactions between *D7Got23* and *D3Rat78* and between *D7Got23* and *D14Mit4*. Results suggested that a modified locus on Chr 7 was mainly responsible for the variance of pigmentary disorder between AGH-*Ednrb*^*sl*^ rats and LEH-*Ednrb*^*sl*^ rats, and two modifier loci showing epistatic interaction may, in part, influence pigment phenotype.

Genetic studies on coat color mutations in mammals have a long history in biomedical research because of their viable and visible phenotypes. Today, a wealth of information about the pathways and genes involved in the pigmentation has been revealed. Nearly 130 genes with approximately 1000 different alleles have been detected to affect coat color^1^. Early in the 19th century, coat color mutation was used to prove Mendel’s laws^2^. Then, coat color mutations were used to generate different inbred lines of visible markers^3^,^4^. Melanin-based pigmentation is highly conserved across vertebrates^5^; thus, color mutations in mammals can provide models for some human diseases. A large number of diseases in humans are associated with pigmentary abnormalities, such as Waardenburg syndrome^6^, Hirschsprung’s disease^7^, oculocutaneous albinism I^8^, and piebaldism^9^. Moreover, the pigmentation system is a classical tool in ecological studies. Selective forces such as aposematism, crypsis, thermoregulation, and sexual signaling drive variation in the pigmentation pattern^10^.

The endothelin3 (Edn3)/endothelin receptor B (Ednrb) ligand–receptor pair is involved in pigmentation^11^,^12^. *Ednrb*-deficient mice exhibit an almost completely white coat, and they develop megacolon^11^. *Ednrb*^*sl*^ is a spontaneous null mutation characterized by deletion of 301 bp in the *Ednrb* gene in rats, resulting in Hirschsprung’s disease and pigmentary disorder^13^. In our previous study, we established two strains with different genetic background but carrying the same *Ednrb*^*sl*^ mutation, namely, AGH-*Ednrb*^*sl*^ and LEH-*Ednrb*^*sl*^^14^; these two strains showed different pigment phenotype. AGH-*Ednrb*^*sl/sl*^ rats showed almost no pigmentation all over the body, whereas a large pigmented spot appeared on the head of LEH-*Ednrb*^*sl/sl*^ rats. Therefore, we hypothesized that modifier loci in the genetic background of LEH modulated the severity of the pigmentary disorder.

In this study, we analyzed the difference in pigmentation between the *Ednrb*^*sl*^-mutated rats; we performed quantitative trait locus (QTL) analysis using the intercross descendants with varying severity of pigmentation disorder, and we tried to search the modifier gene(s) affecting the phenotype.

## Results

### Evaluation of pigmentation in F_2_ *Ednrb* ^*sl/sl*^ pups

**Figure 1:**
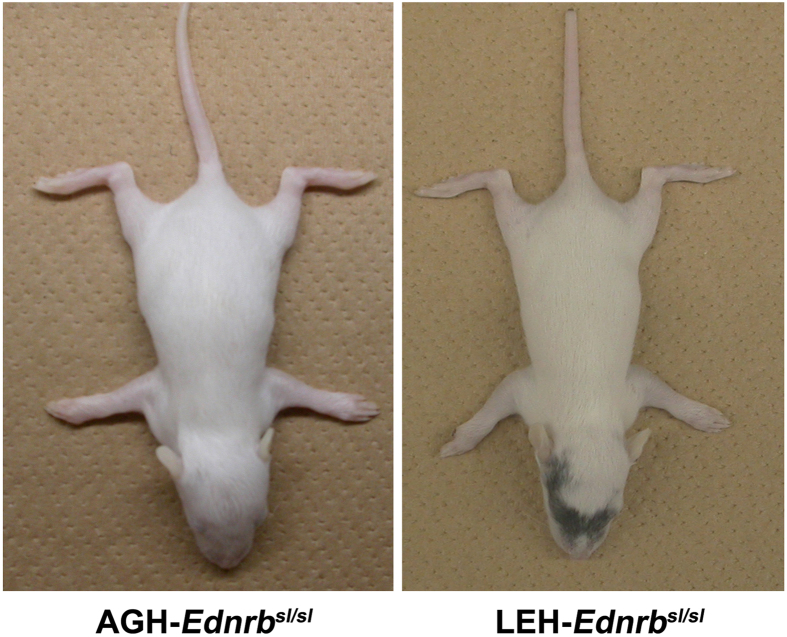
Comparison of the expressivity of the pigment disorder. In 14-day-old rats, no pigmentation on the head was observed in AGH-*Ednrb*^*sl/sl*^ rats, and pigmentation was observed in LEH-*Ednrb*^*sl/sl*^ rats.

**Figure 2:**
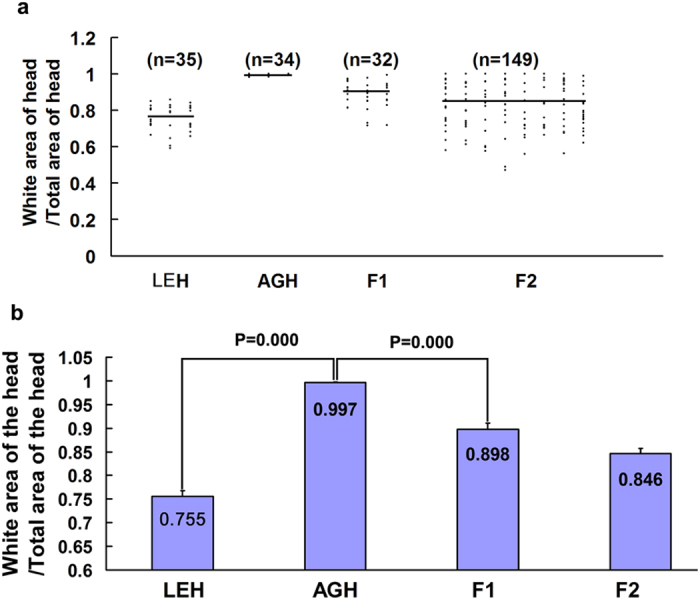
Range of pigment disorder. (**a**) The range of pigment disorder in 14-day-old pups in AGH-*Ednrb*^*sl/sl*^, LEH-*Ednrb*^*sl/sl*^, F_1_, and F_2_. Horizontal lines indicate mean values. (**b**) ANOVA results of the pigment disorder ratio in AGH-*Ednrb*^*sl/sl*^, LEH-*Ednrb*^*sl/sl*^, F_1_, and F_2_. Each bar indicates the mean ± S.E.M.

Homozygous *Ednrb*^*sl/sl*^ rats showed a pigmentary disorder. We reported previously that variations of the pigmentary disorder was observed in *Ednrb*^*sl/sl*^ rats with different genetic backgrounds^14^. AGH-*Ednrb*^*sl/sl*^ rats almost had no pigmentation on their heads, whereas a large pigmented spot on the head was observed in LEH-*Ednrb*^*sl/sl*^ (Fig. 1). We concluded that a modifier locus in the LEH background rescued the pigmentary disturbance to some extent^14^. We calculated the unpigmented coat ratio of AGH, LEH, F_1_, and F_2_ *Ednrb*^*sl/sl*^ rats by using a camera and Photoshop and revealed the degree of variation of the pigmentary disorder. The severity of pigmentary disorder was calculated as a ratio of the unpigmented area in the head (pigment area/total area), which could be used as quantitative trait of the individual. The range of the severity of pigmentary disorder for each homozygous *Ednrb*^*sl/sl*^ rat was presented in a scatter plot. Figure 2a shows that spots for F_2_-*Ednrb*^*sl/sl*^ rats were fairly scattered in the range of two extreme values compared with that of both AGH-*Ednrb*^*sl/sl*^ and LGH-*Ednrb*^*sl/sl*^ rats. Moreover, we calculated the mean value of the pigment disorder ratio for each of the AGH, LEH, F_1_, and F_2_ *Ednrb*^*sl/sl*^ rats, which were 0.997, 0.755, 0.898, and 0.846, respectively (Fig. 2b). The values obtained in AGH-*Ednrb*^*sl/sl*^ rats were highly different from that of LEH-*Ednrb*^*sl/sl*^ rats with significance of P = 0.000, suggesting that the role of modifier(s) in the variance of pigment disorder observed in the LEH strain.

### QTL analysis of the modifiers of pigment disorder in the F_2_ *Ednrb* ^*sl/sl*^ rats

**Figure 3:**
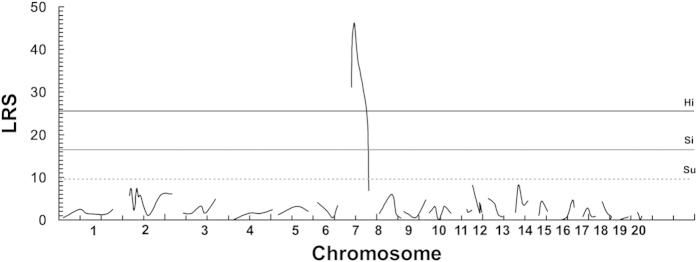
Result of interval mapping by MapManager QTXb20 in F_2_ rats. Analyses of the linkage of the unpigmented area in F_2_ populations to chromosomal loci were performed using the MapManager QTXb20 software. Recombination frequencies (%) were converted into genetic distance (in cM) by using the Kosambi map function; linkage data were provided as LRS scores. Genome-wide significance thresholds were calculated in terms of LRS by carrying out permutation tests for 5,000 permutations. The thresholds for suggestive (Su, LRS = 9.6), significant (Si, LRS = 16.5), and highly significant (Hi, LRS = 25.6) linkages were indicated using dotted, thin, and thick lines, respectively.

**Figure 4:**
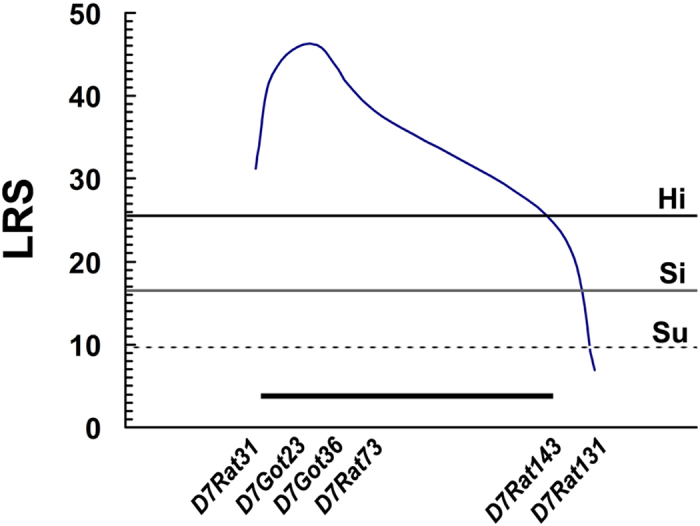
Details of QTL for the severity of pigment disorder on chromosome 7. The QTL on chromosome 7 showed highly significant linkage to the pigment disorder ratio. The dotted, thin, and thick lines represent suggestive (Su, LRS = 9.6), significant (Si, LRS = 16.5), and highly significant (Hi, LRS = 25.6) thresholds, respectively, calculated by 5,000 times permutation tests. The microsatellite markers used for determining genotypes of F_2_ rats are presented along the X-axis. The black bars on the graph indicate approximately 95% confidence intervals.

MapManager QTXb20 software was used for QTL scan of the genome to determine the severity of pigmentary disorder in 149 F_2_-*Ednrb*^*sl/sl*^ rats with 91 microsatellite markers (Table 1), which showed polymorphism between AGH and LEH rats. As many as 5,000 random permutations in 1-centiMorgan (cM) steps were performed for each chromosome to calculate the likelihood ratio statistic (LRS). This LRS can be interpreted as a χ2 statistic or as a LOD score. In addition, the LRS can be converted to the conventional base-10 LOD score by dividing it by 4.61 (twice the natural logarithm of 10)^15^. Results of interval mapping were suggestive, significant, and highly significant linkages, that is, the LRS values were 9.6, 16.5, and 25.6, respectively. A highly significant QTL was detected in the region around *D7Got23* on chromosome (Chr) 7 (Figs 3 and 4), which explained 26% of the total phenotypic variance (Table 2).

Epistatic interaction analysis was performed using the interaction function of MapManager QTXb20. The LRS values of suggestive, significant, and highly significant interactions were 29.2, 38.2, and 49.9, respectively. Three two-locus interactions were identified for pigmentary disorder, namely *D7Got23* (Chr 7) and *D14Mit4* (Chr 14), *D7Got36* (Chr 7) and *D3Rat78* (Chr 3), *D7Rat143* (Chr 7) and *D3Rat78* (Chr 3) (Table 3). We performed ANOVA to confirm the significant epistatic interactions in these microsatellite loci and the vicinal loci. ANOVA results revealed that the *D7Got23* locus showed highly significant interaction with *D3Rat78* locus. Thus, we chose the *D7Got23* locus located at the peak position of the QTL on Chr 7 to represent the loci showing significant epistatic interactions.

### Allelic effects of *Ednrb* ^*sl/sl*^ modifier loci

**Figure 5:**
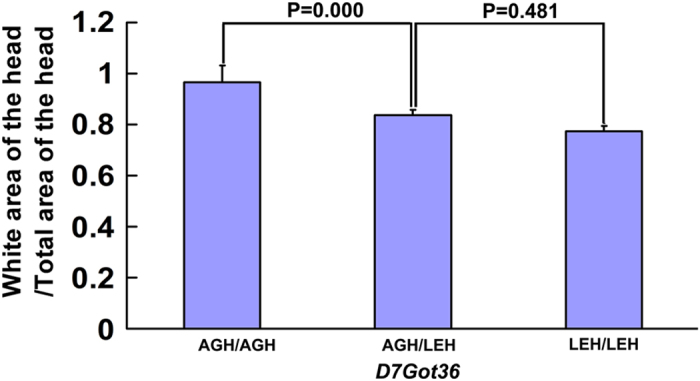
Allelelic effects of *Ednrb*^*sl/sl*^ modifier loci on the severity of pigment disorder. Homozygous *Ednrb*^*slsl*^ genotypes of the 149 experimental rats obtained from the marker closest to the modifier were used to assess the effects of individual loci on the severity of phenotype in F_2_ population. The mean of the pigmentary disorder ratio (white area of the head/total area of the head) is plotted for each genotype class to show the relationship of the number of AGH or LEH alleles to the ratio of pigment disorder for this locus. Markers used to generate genotype information are listed beneath the plot. Genotype groups are defined as AGH/AGH, AGH/LEH, and LEH/LEH. Each bar indicates the mean ± S.E.M.

Modifier loci influence the phenotype but cannot revert the effects of a predisposed mutation such as *Ednrb*^*sl/sl*^^16^. To estimate the effects of a modifier locus on the pigmentary disorder in *Ednrb*^*sl/sl*^ individuals, a complete evaluation of the genotypic information in all F_2_-*Ednrb*^*sl/sl*^ progenies was performed. Figure 5 shows the extent of pigment disorder that was evidently modified by the LEH alleles at the modifier locus on Chr 7. The ratio of pigmentary disorder was higher in homozygous AGH alleles compared with that in heterozygotes or homozygotes for LEH alleles. Significant difference was found between the ratios of homozygous AGH alleles and heterozygotes (P = 0.000), whereas no significant difference (P = 0.481) was found between heterozyotes and homozygous LEH alleles. Thus, we considered that the effect of LEH allele was approximately dominant.

## Discussion

Coat color generally depends on the amount of melanin produced by melanocytes derived from neural crest cells^17^,^18^. Endothelin receptor B (EDNRB) is a G-protein-coupled receptor with seven transmembrane domains that is necessary during the development of neural crest and melanocytes^19^. Mouse with null *Ednrb* gene appeared *albino*^11^. However, *Ednrb*^*sl/sl*^ rats with different genetic background showed variation in their extent of pigmentation^14^. More severe *albino* phenotype was observed in AGH-*Ednrb*^*sl/sl*^ strain, whereas the pigmentation disturbance was reverted to some extent in LEH-*Ednrb*^*sl/sl*^ rats (Fig. 1). This observation suggested that the modifier loci in the LEH allele affected the phenotype.

**Figure 6:**
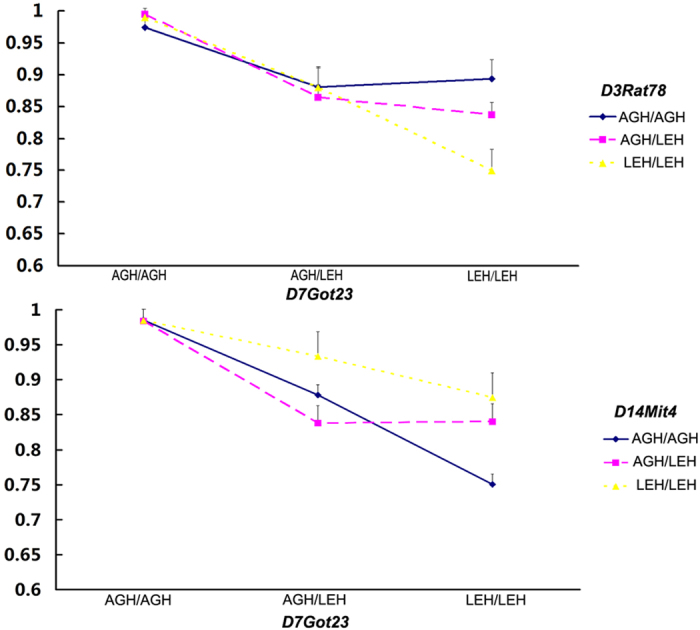
Highly significant epistatic interactions influence the extent of pigment disorder in F_2_ rats. Two highly significant epistatic interactions were detected between *D3Rat78* locus and *D7Got23* locus (**a**) and between *D14Mit4* locus and *D7Got23* locus (**b**). Each bar indicates the mean ±S.E.M.

We performed a genome-wide scan in F_2_ progenies (AGH-*Ednrb*^*sl/sl*^×LEH-*Ednrb*^*sl/sl*^) to examine the effect of the modifier loci in pigment disorder in rats using QTL analysis. Results revealed a highly significant QTL around *D7Got23* on Chr 7 (Fig. 3); it has an extremely high LRS value and has 26% contribution to the total phenotypic variance (Table 2). Therefore, we assumed that one or more modifier genes responsible for pigment disorder may be located in the same region in Chr 7. The LEH allele in the modifier locus on Chr 7 increased the extent of the pigmented area (Fig. 5). Highly significant difference was observed between the ratios of homozygous AGH alleles and heterozygotes indicating that LEH allele was dominant (Fig. 5). While significant difference was detected between the ratios of homozygous LEH alleles and heterozygotes revealing other loci could influence pigment phenotype as well. Furthermore, a synteny analysis in other mammals revealed that the QTL around *D7Got23* locus on Chr 7 in rats corresponded comparatively to a region on Chr 10 in mouse, which housed an *Ednrb*^*s*^ modifier locus (*k10*) determining the expressivity of a white forelock and dorsal hypopigmentation^20^. This result suggested that *D7Got23* locus is a region of conserved synteny between rat and mouse genomes. In addition, *D7Got23* locus showed epistatic interactions with *D14Mit4* locus and *D3Rat78* locus. When *D7Got23* locus had LEH-homozygous genotype, the LEH-homozygous genotype of *D3Rat78* locus showed the highest extent of pigmented coat in F_2_ progenies, followed by AGH/LEH-heterozygous and then the AGH-homozygous genotypes (Fig. 6). The LEH allele resisted the pigmentary disorder in both *D7Got23* and *D3Rat78* loci. We noted that the physical position of *D14Mit4* (43.8 Mb, RGSC 5.0) is close to that of *D14Got40* (35.3 Mb, RGSC 5.0), which was detected as the hooded locus^21^. The hooded phenotype is one of coat color phenotypes in rat with many alleles causing different extents of pigmented coat area^22^,^23^,^24^. Therefore, the modifier locus we detected for the pigmentary disorder on Chr 7 might show epistatic interactions with the hooded locus. Interestingly, the effects of *D7Got23* locus and *D14Mit4* locus seemed to be opposite. LEH allele of *D7Got23* locus resisted the pigmentary disorder, while that of *D14Mit4* locus increased the extent of the disorder (Fig. 6). The *D7Got23* locus was the main one responsible for the variation of pigmentary disorder. The *D14Mit4* locus showed significant effect on the pigmentary phenotype when the *D7Got23* locus was homozygous for LEH allele. The F_2_ rats owning the LEH-homozygous genotype at the *D7Got23* locus and the AGH-homozygous genotype at the *D14Mit4* locus showed the highest extent of pigmented coat.

Possible modifier genes responsible for the pigmentary disorder within the identified chromosomal region were identified using some bioinformatics methods, such as genome annotation combined with literature searches to check the confidence interval of the QTL on Chr 7 for potential genes that might be involved in the development of melanocytes^25^. More than 70 genes were identified (Supplementary Table S1). Among these candidates, *Lgr5* and *Wif1* attracted our attention. Both of them are associated with *WNT* signal pathway, which is responsible for the development of melanocytes^26^,^27^. Mass spectrometry demonstrated that Lgr4 and Lgr5 associate with the Frizzled/Lrp Wnt receptor complex^28^. However, the relationship between *Lgr5* gene and pigmentation was unknown. The *Wif1* gene, a Wnt inhibitory factor 1, was expressed not only in the melanocytes of normal human skin but also in cultured melanocytes and promoted melanogenesis in normal melanocytes^29^. In addition, we sequenced the coding region of these genes; however, we failed to find any difference between AGH and LEH strains. The causal difference might exist within the regulatory region of *Lgr5 or Wif1* gene. But it was possible that other causative genes had not been sequenced in our study. The main reason that we failed to identify the modifier gene(s) was the broad confidence interval of the QTL on Chr 7 (between *D7Rat31* and *D7Rat143*, from 28.5 Mb to 79.0 Mb, RGSC 3.4). We tried hard to find more markers showing polymorphism between AGH and LEH strains in this interval to improve the confidence interval, but we found none. The origin of this interval in AGH and LEH strains might be the same. In addition, the low number of the F_2_ rats limited the precision of the result. To elucidate the pigment disorder, other biological methods must be employed to identify the modifier gene(s) in this region.

In conclusion, we have identified a highly significant QTL on Chr 7 by using two pigmentary disorder strains. Other two loci, namely, *D14Mit4* locus on Chr 14 and *D3Rat78* locus on Chr 3, show interaction with the main QTL on Chr 7 and may synergistically affect pigmentation.

## Methods

### Animals

The F_1_ family was produced by crossing 2 AGH/Hkv (aganglionosis Hokkaido)-*Ednrb*^*sl*/+14^ males and 8 LEH/Hkv (Long-Evans Hokkaido)-*Ednrb*^*sl*/+14^ females. Five heterozygous males and 20 heterozygous females of F_1_ rats were bred to generate the F_2_ animals (*n* = 592), from which 149 *Ednrb*^*sl/sl*^ pups were selected by genotyping through PCR amplification using a pair of specific primers (F-CCTCCTGGACTAGAGGTTCC and R-ACGACTTAGAAAGCTACACT) and then flanking the site of deletion with 301 bp in *Ednrb* gene. PCR products were electrophoresed in 1.5% agarose gels to distinguish the wild (511 bp) type and the mutant (210 bp) type. AGH (*n* = 35), LEH (*n* = 34), and F_1_ rats (*n* = 32) were raised for the determination of the severity of pigment disorder in each strain. Animals were maintained in specific pathogen-free conditions, fed, and supplied with water *ad libitum*. All research and experimental protocols were in accordance with the Regulation for the Care and Use of Laboratory Animals, Hokkaido University and approved by the President of Hokkaido University following to the review of the Institutional Animal Care and Use Committee (Approval ID: No. 110226).

### Measurement of unpigmented coat ratio

Photographs from the dorsal side of the rats were taken with a COOLPIX 4500 digital camera (Nikon, Tokyo, Japan). The total area of the head and of the pigmented area in the head were traced with lasso tools and calculated as pixels using the histogram function of Photoshop Elements 4.0 (Adobe Systems, California, USA). The ratio of unpigmented area of the head (1-pigmented area/total area) was then calculated as the phenotypic value of pigment disorder.

### Microsatellite genotyping

A total of 149 intercross progenies were selected for the genome-wide scan. Extraction of genomic DNA from the tail clips was performed using the standard methods. A total of 91 polymorphic microsatellite markers covering all 20 autosomes were selected from the National Center for Biotechnology Information <NCBI; http://www.ncbi.nlm.nih.gov/> (RGSC 5.0) for the genome-wide scan at 10–30 Mbp resolution (Table 1). The X chromosome was not scanned in our study because gender bias of pigment phenotype was not found in F_2_ rats. The PCR procedure is described as follows: each 10 μL reaction contains 0.5 μL (10 ng) DNA, 0.5 μL (5 pmol) of each primer, 5.5 μL 2 × Taq MasterMix (CWBIO, Beijing, China), and 3.0 μL ddH_2_O. Touchdown-PCR was performed as follows: 2 min at 95 °C, followed by 10 cycles for 30 s at 95 °C, 30 s at 63–54 °C, 30 s at 72 °C, then 25 cycles for 30 s at 95 °C, 30 s at 54 °C, 30 s at 72 °C, and a final extension at 72 °C for 10 min. PCR products were electrophoresed using 10% acrylamide gels at 160 V for 1.5–3 h, stained with ethidium bromide, and photographed under an ultraviolet lamp (Supplementary Fig. S1).

### Linkage analysis

Genotyping results and phenotypic values were analyzed using MapManager QTXb20 to identify the pigment disorder modifier loci^30^. A maximum likelihood algorithm with interval mapping was performed to examine linkage probability. Permutation tests were done in 1-cM steps for 5,000 permutations to determine the suggestive, significant, or highly significant levels of statistics. Interactions between all pairs of markers were also screened using MapManager QTXb20 program. We used 5,000 permutations in 1-cM steps to detect possible interactions using regression.

### Two-locus interaction analysis

Interaction effects or epistasis of all pairs of marker loci were tested with the interaction function of Map Manager QTXb20^15^. According to the manual, pairs of loci had to pass two tests to claim significant interaction effects. First, the total effect of the two loci had a *p*-value < 10^−5^. Second, the interaction effects itself had a *p*-value < 0.01.

### Statistical analyses

Statistical analyses was perforemed using the SPSS 19 software and R. Significance test for pigment disorder ratio in AGH-*Ednrb*^*sl/sl*^, LEH-*Ednrb*^*sl/sl*^, F_1_, and F_2_, as well as in three types of alleles of *D7Got23* located on the peak were analyzed using ANOVA in SPSS 19. Significance test for the corresponding phenotypic mean values of nine types of alleles of two pairs loci showing epistatic interactions were analyzed by ANOVA in R.

## Additional Information

**How to cite this article**: Huang, J. *et al.* QTL analysis of modifiers for pigmentary disorder in rats carrying *Ednrb^sl^* mutations. *Sci. Rep.* **6**, 19697; doi: 10.1038/srep19697 (2016).

## Supplementary Material

Supplementary Information

## Figures and Tables

**Table 1:** Microsatellite markers used for genotyping F_2_ intercrossed progenies.

Microsatellite Markers	Position (Mbp)	Microsatellite Markers	Position (Mbp)	Microsatellite Markers	Position (Mbp)	Microsatellite Markers	Position (Mbp)
*D1Rat392*	22	*D4Rat183*	187	*D9Rat153*	107	*D14Rat94*	89
*D1Rat343*	99	*D4Rat204*	243	*D10Mgh27*	12	*D15Rat5*	25
*D1Rat269*	133	*D5Rat125*	22	*D10Rat217*	17	*D15Rat6*	37
*D1Rat159*	218	*D5Rat196*	107	*D10Rat177*	29	*D15Rat48*	66
*D1Got225*	255	*D5Rat44*	162	*D10Got60*	40	*D16Rat78*	21
*D2Rat252*	42	*D6Got15*	30	*D10Rat163*	50	*D16Rat3*	44
*D2Rat116*	52	*D6Got74*	71	*D10Mit2*	65	*D16Got63*	69
*D2Mgh14*	62	*D6Rat165*	103	*D10Rat154*	75	*D16Rat55*	78
*D2Rat201*	69	*D6Rat11*	124	*D10Rat7*	104	*D17Rat2*	68
*D2Mit33*	73	*D7Rat31*	32	*D11Got45*	67	*D17Rat12*	29
*D2Rat385*	79	*D7Got23*	36	*D11Rat63*	72	*D17Rat24*	50
*D2Mit5*	86	*D7Got36*	47	*D11Rat43*	90	*D17Rat175*	87
*D2Rat21*	95	*D7Rat73*	61	*D12Rat58*	1	*D18Rat132*	26
*D2Rat123*	132	*D7Rat143*	105	*D12Rat76*	34	*D18Rat34*	48
*D2Got114*	191	*D7Rat131*	115	*D12Rat14*	34	*D18Got63*	68
*D2Rat241*	243	*D8Rat68*	21	*D12Rat86*	46	*D18Rat86*	66
*D3Rat57*	8	*D8Rat33*	79	*D13Rat150*	21	*D19Rat15*	27
*D3Mgh7*	45	*D8Rat18*	99	*D13Rat149*	50	*D19Rat27*	30
*D3Rat34*	89	*D8Rat8*	121	*D13Rat180*	67	*D19Got53*	62
*D3Rat287*	111	*D9Got6*	4	*D13Rat131*	88	*D20Mit4*	34
*D3Rat78*	159	*D9Rat41*	14	*D14Got35*	29	*D20Rat55*	46
*D4Mgh16*	61	*D9Got27*	20	*D14Mit4*	44	*D20Got47*	52
*D4Rat26*	135	*D9Mit3*	63	*D14Rat45*	70		

**Table 2:** Characteristics of QTLs detected for variance of pigment disorder in F_2_ intercrossed progenies.

Chr	Locus	Position (Mbp)	LRS	%	P	CI	Add
Chr 2	*D2Rat116*	52	7.3	5	0.02576	74	0.04
Chr 2	*D2Mit33*	73	6.1	4	0.04794	89	0.02
Chr 2	*D2Rat385*	79	7.4	5	0.02431	73	0.02
Chr 7	*D7Rat31*	32	30.0	18	0.00000	19	−0.08
Chr 7	*D7Got23*	36	45.0	26	0.00000	14	−0.10
Chr 7	*D7Got36*	47	44.0	26	0.00000	14	−0.10
Chr 7	*D7Rat73*	61	34.6	21	0.00000	17	−0.08
Chr 7	*D7Rat143*	105	24.6	15	0.00000	23	−0.07
Chr 12	*D12Rat58*	1	8.1	5	0.01759	67	0.03
Chr 14	*D14Mit4*	11	8.2	5	0.01681	67	0.03

%: Percentage of total variance attributable to locus. CI: 95% confidence interval of QTL location as calculated by QTX software. Add: Addictive effect of the allele from LEH strain compared with that from AGH strain.

**Table 3:** Interaction results.

Chr 1	Locus 1	Position 1 (Mbp)	Chr 2	Locus 2	Position 2 (Mbp)	LRS	P	IX	Main 1	% 1	Main 2	% 2
Chr 7	*D7Got23*	36	Chr 14	*D14Mit4*	11	68.6	0.00000	13.6	45.0	26	8.2	5
Chr 7	*D7Got36*	47	Chr 3	*D3Rat78*	159	65.0	0.00000	17.7	44.0	26	4.9	3
Chr 7	*D7Rat143*	105	Chr 3	*D3Rat78*	159	45.8	0.00000	14.2	24.6	15	4.9	3

LRS: Total LRS for association; IX: Interaction LRS; Main 1: LRS for locus 1 main effect; Main 2: LRS for locus 2 main effect; % 1: Percentage of total variance attributable to locus 1; % 2: Percentage of total variance attributable to locus 2.
