# Supplementary material for: QTL analysis of modifiers for pigmentary disorder in rats carrying Ednrbsl mutations
Source: Sci Rep. 2016 Jan 22;6:19697. doi: 10.1038/srep19697 (PMC4726237; doi:10.1038/srep19697)
Supplement: Supplementary Information [file srep19697-s1.pdf]

QTL analysis of modifiers for pigmentary disorder in rats carrying *Ednrb*^{sl} mutations

Jieping Huang[1,2§], Ruihua Dang[2§*], Daisuke Torigoe[3,5], Anqi Li[2], Chuzhao Lei[2*], Nobuya Sasaki[4], Jinxi Wang[3], and Takashi Agui[3]

*To whom correspondence should be addressed. Email: dangruihua@nwsuaf.edu.cn (RD); leichuzhao1118@126.com (CL)

This PDF file includes:
Supplementary Figure S1
Supplementary Table S1

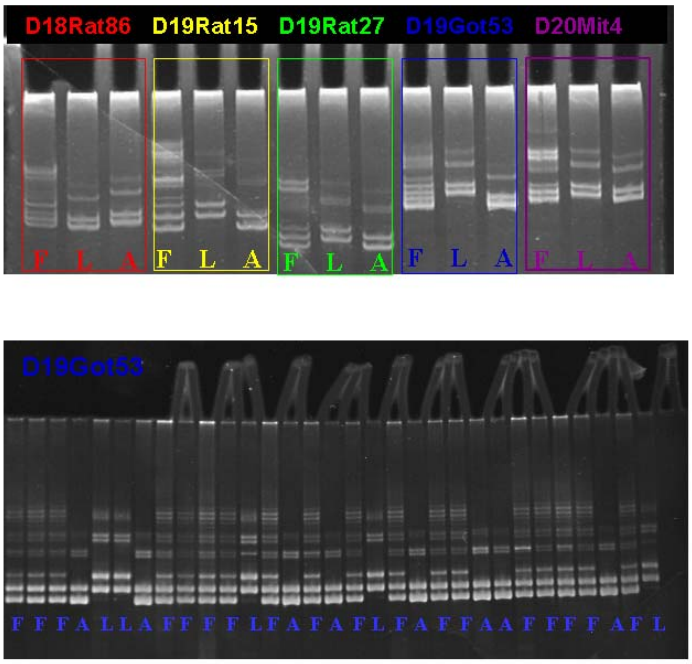

Figure S1. **Part of polymorphic microsatellite markers and microsatellite genotyping.** Microsatellite markers showing polymorphisms among AGH rats, LEH rats, and F_1 rats were selected (up). AGH rats, LEH rats, and F_1 rats were used as controls for Microsatellite genotyping (down). "F", "L", and "A" represented heterozygote, LEH homozygote, and AGH homozygote, respectively.

Table S1. Possible modifier genes responsible for the pigmentary disorder within the identified chromosomal region.

P value	Object set1	Symbol1	Name1	Position1	Object set2	Symbol2	Name2	Position2
1.51E-40668	Rat Gene	Ifng	interferon gamma	Rn:7:57642485-57646522	Mouse Gene	Ifng	interferon gamma	Mm:10:117878103-117882948
6.37E-40330	Rat Gene	Nts	neurotensin	Rn:7:40494497-40495295	Mouse Gene	Nts	neurotensin	Mm:10:101944390-101953052
6.55E-40042	Rat Gene	Mdm2	transformed mouse 3T3 cell double minute 2	Rn:7:57018654-57054110	Mouse Gene	Mdm2	transformed mouse 3T3 cell double minute 2	Mm:10:117125944-117147814
1.30E-40021	Rat Gene	Plxnc1	plexin C1	Rn:7:32007444-32157845	Mouse Gene	Plxnc1	plexin C1	Mm:10:94255931-94407212
2.92E-40020	Rat Gene	Mtdh	metadherin	Rn:7:69547911-69603745	Human Gene	MTDH	metadherin	Hs:8:98656407-98742488
2.92E-40020	Rat Gene	Syt1	synaptotagmin I	Rn:7:47261066-47815950	Mouse Gene	Syt1	synaptotagmin I	Mm:10:107934706-108448038
2.92E-40020	Rat Gene	Stat6	signal transducer and activator of transcription 6	Rn:7:67622590-67662987	Mouse Gene	Stat6	signal transducer and activator of transcription 6	Mm:10:127080042-127098013
5.90E-40018	Rat Gene	Dusp6	dual specificity phosphatase 6	Rn:7:36913534-36917764	Mouse Gene	Dusp6	dual specificity phosphatase 6	Mm:10:98725865-98730118
5.90E-40018	Rat Gene	Dcn	decorin	Rn:7:35029274-35065458	Mouse Gene	Dcn	decorin	Mm:10:96945001-96980785
5.90E-40018	Rat Gene	Gli1	GLI-Kruppel family member GLI1	Rn:7:67311850-67313540	Mouse Gene	Gli1	GLI-Kruppel family member GLI1	Mm:10:126766938-126778635
5.90E-40018	Rat Gene	Phlda1	pleckstrin homology-like domain, family A, member 1	Rn:7:50561474-50563217	Mouse Gene	Phlda1	pleckstrin homology-like domain, family A, member 1	Mm:10:110943460-110945484
5.90E-40018	Rat Gene	Wif1	Wnt inhibitory factor 1	Rn:7:60417062-60421426	Mouse Gene	Wif1	Wnt inhibitory factor 1	Mm:10:120471016-120537706
5.90E-40018	Rat Gene	Irak3	interleukin-1 receptor-associated kinase 3	Rn:7:59429927-59488412	Mouse Gene	Irak3	interleukin-1 receptor-associated kinase 3	Mm:10:119578704-119639186
5.93E-40018	Rat Gene	Sdc2	syndecan 2	Rn:7:68311497-68426917	Mouse Gene	Sdc2	syndecan 2	Mm:15:32850478-32964485
5.42E-20414	Rat Gene	Tph2	tryptophan hydroxylase 2	Rn:7:54341665-54451339	Mouse Gene	Tph2	tryptophan hydroxylase 2	Mm:10:114515697-114622078
1.10E-20388	Rat Gene	Avpr1a	arginine vasopressin receptor 1A	Rn:7:62246266-62250191	Mouse Gene	Avpr1a	arginine vasopressin receptor 1A	Mm:10:121885555-121890509
1.14E-20293	Rat Gene	Lum	lumican	Rn:7:35103750-35110554	Mouse Gene	Lum	lumican	Mm:10:97028464-97035338
2.51E-20184	Rat Gene	Myf5	myogenic factor 5	Rn:7:46210584-46212529	Mouse Gene	Myf5	myogenic factor 5	Mm:10:106919964-106923190
1.01E-20112	Rat Gene	Ddit3	DNA-damage inducible transcript 3	Rn:7:67268487-67273294	Mouse Gene	Ddit3	DNA-damage inducible transcript 3	Mm:10:126727830-126733342
2.49E-20072	Rat Gene	Lrp1	low density lipoprotein receptor-related protein 1	Rn:7:67541343-67622669	Mouse Gene	Lrp1	low density lipoprotein receptor-related protein 1	Mm:10:126975217-127058204
8.20E-20069	Rat Gene	Kera	keratocan	Rn:7:35142278-35148859	Mouse Gene	Kera	keratocan	Mm:10:97069839-97076322
6.63E-20062	Rat Gene	Lgr5	leucine rich repeat containing G protein coupled receptor 5	Rn:7:54758873-54897394	Mouse Gene	Lgr5	leucine rich repeat containing G protein coupled receptor 5	Mm:10:114887367-115024836
9.87E-20061	Rat Gene	Frs2	fibroblast growth factor receptor substrate 2	Rn:7:56435173-56446435	Mouse Gene	Frs2	fibroblast growth factor receptor substrate 2	Mm:10:116507185-116585530
5.54E-20051	Rat Gene	Lta4h	leukotriene A4 hydrolase	Rn:7:30502483-30535333	Mouse Gene	Lta4h	leukotriene A4 hydrolase	Mm:10:92916156-92947619
4.80E-20047	Rat Gene	Eea1	early endosome antigen 1	Rn:7:33311103-33413833	Mouse Gene	Eea1	early endosome antigen 1	Mm:10:95403297-95508152
7.85E-20045	Rat Gene	Ppp1r12a	protein phosphatase 1, regulatory (inhibitor) subunit 12A	Rn:7:46896822-47006293	Mouse Gene	Ppp1r12a	protein phosphatase 1, regulatory (inhibitor) subunit 12A	Mm:10:107599456-107714631
7.07E-20042	Rat Gene	Trhde	thyrotropin-releasing hormone degrading enzyme	Rn:7:53612156-54024183	Mouse Gene	Trhde	TRH-degrading enzyme	Mm:10:113835879-114239363
1.85E-20035	Rat Gene	Pawr	PRKC, apoptosis, WT1, regulator	Rn:7:47060432-47139881	Mouse Gene	Pawr	PRKC, apoptosis, WT1, regulator	Mm:10:107769245-107851447
1.17E-20033	Rat Gene	Tac2	tachykinin 2	Rn:7:67737797-67744404	Mouse Gene	Tac2	tachykinin 2	Mm:10:127161534-127168823
1.23E-20032	Rat Gene	Cpm	carboxypeptidase M	Rn:7:56952660-57008965	Mouse Gene	Cpm	carboxypeptidase M	Mm:10:117066556-117124408
1.58E-20029	Rat Gene	Klf10	Kruppel-like factor 10	Rn:7:73842010-73847864	Mouse Gene	Klf10	Kruppel-like factor 10	Mm:15:38224171-38230461
2.67E-20029	Rat Gene	Geft	RhoA/RAC/CDC42 exchange factor	Rn:7:67158181-67165211	Mouse Gene	Arhgef25	Rho guanine nucleotide exchange factor (GEF) 25	Mm:10:126619585-126627075
3.03E-20029	Rat Gene	Cradd	CASP2 and RIPK1 domain containing adaptor with death domain	Rn:7:32379423-32536642	Mouse Gene	Cradd	CASP2 and RIPK1 domain containing adaptor with death domain	Mm:10:94637899-94786199
3.03E-20029	Rat Gene	Fzd6	frizzled homolog 6 (Drosophila)	Rn:7:74608138-74610532	Mouse Gene	Fzd6	frizzled homolog 6 (Drosophila)	Mm:15:38837898-38869732
6.85E-20027	Rat Gene	Myf6	myogenic factor 6	Rn:7:46220242-46222138	Mouse Gene	Myf6	myogenic factor 6	Mm:10:106929916-106931785
3.83E-20024	Rat Gene	Ywhaz	tyrosine 3-monooxygenase/tryptophan 5-monooxygenase activation protein, zeta polypeptide	Rn:7:72304554-72327161	Mouse Gene	Ywhaz	tyrosine 3-monooxygenase/tryptophan 5-monooxygenase activation protein, zeta polypeptide	Mm:15:36700525-36724302
1.23E-20021	Rat Gene	Caps2	calcyphosphine 2	Rn:7:51212317-51258552	Mouse Gene	Caps2	calcyphosphine 2	Mm:10:111600677-111653611
1.30E-20021	Rat Gene	B4galnt1	beta-1,4-N-acetyl-galactosaminyl transferase 1	Rn:7:67141511-67148426	Mouse Gene	B4galnt1	beta-1,4-N-acetyl-galactosaminyl transferase 1	Mm:10:126602281-126609386
1.11E-20020	Rat Gene	Rab21	RAB21, member RAS oncogene family	Rn:7:54570880-54595665	Mouse Gene	Rab21	RAB21, member RAS oncogene family	Mm:10:114726919-114752647
2.68E-20020	Rat Gene	Epyc	epiphycan	Rn:7:35184757-35218169	Mouse Gene	Epyc	epiphycan	Mm:10:97106702-97145088
2.88E-20020	Rat Gene	Osr2	odd-skipped related 2 (Drosophila)	Rn:7:70789852-70796977	Mouse Gene	Osr2	odd-skipped related 2	Mm:15:35225867-35233060
2.92E-20020	Rat Gene	Cohh1	Cohen syndrome homolog 1	Rn:7:70860765-71486400	Mouse Gene	Vps13b	vacuolar protein sorting 13B (yeast)	Mm:15:35301304-35859849
2.92E-20020	Rat Gene	Cand1	cullin associated and neddylation disassociated 1	Rn:7:58481934-58519822	Mouse Gene	Cand1	cullin associated and neddylation disassociated 1	Mm:10:118636311-118677111
2.92E-20020	Rat Gene	Dpys	dihydropyrimidinase	Rn:7:75449118-75466890	Mouse Gene	Dpys	dihydropyrimidinase	Mm:15:39600031-39689016
4.61E-20020	Rat Gene	Rims2	regulating synaptic membrane exocytosis 2	Rn:7:74777732-75292501	Mouse Gene	Rims2	regulating synaptic membrane exocytosis 2	Mm:15:39029878-39513486
3.78E-20019	Rat Gene	Ptprr	protein tyrosine phosphatase, receptor type, R	Rn:7:55344975-55633672	Mouse Gene	Ptprr	protein tyrosine phosphatase, receptor type, R	Mm:10:115455269-115711988
4.76E-20019	Rat Gene	Nab2	Ngfi-A binding protein 2	Rn:7:67663919-67669962	Mouse Gene	Nab2	Ngfi-A binding protein 2	Mm:10:127097974-127105624
1.86E-20018	Rat Gene	Hal	histidine ammonia lyase	Rn:7:30541183-30571416	Mouse Gene	Hal	histidine ammonia lyase	Mm:10:92951513-92982049
5.90E-20018	Rat Gene	Dctn2	dynactin 2	Rn:7:67244622-67259886	Mouse Gene	Dctn2	dynactin 2	Mm:10:126703455-126718862
5.90E-20018	Rat Gene	Baalc	brain and acute leukemia, cytoplasmic	Rn:7:74446996-74520280	Mouse Gene	Baalc	brain and acute leukemia, cytoplasmic	Mm:15:38765455-38782810
5.90E-20018	Rat Gene	Grip1	glutamate receptor interacting protein 1	Rn:7:59170845-59326858	Mouse Gene	Grip1	glutamate receptor interacting protein 1	Mm:10:118891090-119524317
5.90E-20018	Rat Gene	Azin1	antizyme inhibitor 1	Rn:7:74179861-74210177	Mouse Gene	Azin1	antizyme inhibitor 1	Mm:15:38417185-38449021
5.90E-20018	Rat Gene	Tm7sf4	transmembrane 7 superfamily member 4	Rn:7:75349840-75357112	Mouse Gene	Dcstamp	dentrocyte expressed seven transmembrane protein	Mm:15:39577478-39592480
5.90E-20018	Rat Gene	Slc16a7	solute carrier family 16 (monocarboxylic acid transporters), member 7	Rn:7:65230721-65390741	Mouse Gene	Slc16a7	solute carrier family 16 (monocarboxylic acid transporters), member 7	Mm:10:124664541-124765591
5.90E-20018	Rat Gene	Btg1	B-cell translocation gene 1, anti-proliferative	Rn:7:34049570-34051828	Mouse Gene	Btg1	B cell translocation gene 1, anti-proliferative	Mm:10:96079661-96082261
6.12E-20018	Rat Gene	Myo1a	myosin IA	Rn:7:67722688-67731600	Mouse Gene	Myo1a	myosin IA	Mm:10:127142312-127157994
6.77E-20016	Rat Gene	Dd5	progestin induced protein	Rn:7:73488424-73512532	Mouse Gene	Ubr5	ubiquitin protein ligase E3 component n-recognin 5	Mm:15:37897083-37978898
9.37E-20016	Rat Gene	Tspan31	tetraspanin 31	Rn:7:67039898-67042772	Mouse Gene	Tspan31	tetraspanin 31	Mm:10:126504346-126507317
2.74E-20015	Rat Gene	Zfpm2	zinc finger protein, multitype 2	Rn:7:76324843-76675169	Mouse Gene	Zfpm2	zinc finger protein, multitype 2	Mm:15:40486588-40936138
1.07E-20014	Rat Gene	E2f7	E2F transcription factor 7	Rn:7:49710871-49771412	Mouse Gene	E2f7	E2F transcription factor 7	Mm:10:110182521-110224440
2.18E-20013	Rat Gene	Centg1	centaurin, gamma 1	Rn:7:67052029-67059503	Mouse Gene	Agap2	ArfGAP with GTPase domain, ankyrin repeat and PH domain 2	Mm:10:126515963-126530225
3.32E-20013	Rat Gene	Rrm2b	ribonucleotide reductase M2 B (TP53 inducible)	Rn:7:73452888-73480282	Mouse Gene	Rrm2b	ribonucleotide reductase M2 B (TP53 inducible)	Mm:15:37853707-37891073
6.63E-20013	Rat Gene	Ptprb	protein tyrosine phosphatase, receptor type, B	Rn:7:55634330-55732082	Mouse Gene	Ptprb	protein tyrosine phosphatase, receptor type, B	Mm:10:115738562-115820989
1.03E-20012	Rat Gene	Arhgap9	Rho GTPase activating protein 9	Rn:7:67302835-67309117	Mouse Gene	Arhgap9	Rho GTPase activating protein 9	Mm:10:126760783-126766999
1.03E-20012	Rat Gene	Tmem5	transmembrane protein 5	Rn:7:61878670-61891690	Mouse Gene	Tmem5	transmembrane protein 5	Mm:10:121515170-121534427
1.45E-20012	Rat Gene	Kcnc2	potassium voltage gated channel, Shaw-related subfamily, member 2	Rn:7:51347043-51508400	Mouse Gene	Kcnc2	potassium voltage gated channel, Shaw-related subfamily, member 2	Mm:10:111708179-111903360
1.43E-20011	Rat Gene	Pop1	processing of precursor 1, ribonuclease P/MRP family, (S. cerevisiae)	Rn:7:69965163-70000525	Mouse Gene	Pop1	processing of precursor 1, ribonuclease P/MRP family, (S. cerevisiae)	Mm:15:34425059-34460403
1.89E-20011	Rat Gene	Tspyl5	testis-specific protein, Y-encoded-like 5	Rn:7:69082672-69096515	Human Gene	TSPYL5	TSPY-like 5	Hs:6:116571124-116575321
5.26E-20011	Rat Gene	Ptdss1	phosphatidylserine synthase 1	Rn:7:68009131-68082922	Mouse Gene	Ptdss1	phosphatidylserine synthase 1	Mm:13:67034359-67098944
9.75E-20011	Rat Gene	Stk3	serine/threonine kinase 3 (STE20 homolog, yeast)	Rn:7:70447334-70618175	Mouse Gene	Stk3	serine/threonine kinase 3	Mm:15:34805251-35085561
1.03E-20010	Rat Gene	Rassf3	Ras association (RalGDS/AF-6) domain family member 3	Rn:7:60742406-60804477	Mouse Gene	Rassf3	Ras association (RalGDS/AF-6) domain family member 3	Mm:10:120847406-120913306
